# Fracture Resistance of a Two-Piece Zirconia Implant System after Artificial Loading and/or Hydrothermal Aging—An In Vitro Investigation

**DOI:** 10.3390/jfb14120567

**Published:** 2023-12-15

**Authors:** Ralf-Joachim Kohal, Tim Schikofski, Erik Adolfsson, Kirstin Vach, Sebastian Berthold Maximilian Patzelt, Julian Nold, Gregor Wemken

**Affiliations:** 1 Medical Center—University of Freiburg, Center for Dental Medicine, Department of Prosthetic Dentistry, Faculty of Medicine, University of Freiburg, 79106 Freiburg, Germany; tim.schikofski@students.uni-freiburg.de (T.S.); julian.nold@uniklinik-freiburg.de (J.N.); gregor.wemken@uniklinik-freiburg.de (G.W.); 2 RISE Research Institutes of Sweden, 431 53 Mölndal, Sweden; erik.adolfsson@ri.se; 3 Medical Center—University of Freiburg, Institute for Medical Biometry and Statistics, Faculty of Medicine, University of Freiburg, 79104 Freiburg, Germany; kirstin.vach@uniklinik-freiburg.de; 4 Private Dental Clinic, 78658 Zimmern ob Rottweil, Germany

**Keywords:** zirconia oral implants, loading, aging

## Abstract

The purpose of the present study was to assess the fracture resistance of a two-piece alumina-toughened zirconia implant system with a carbon-reinforced PEEK abutment screw. Methods: Thirty-two implants with screw-retained zirconia abutments were divided into four groups of eight samples each. Group 0 (control group) was neither loaded nor aged in a chewing simulator; group H was hydrothermally aged; group L was loaded with 98 N; and group HL was subjected to both hydrothermal aging and loading in a chewing simulator. One sample of each group was evaluated for *t-m* phase transformation, and the others were loaded until fracture. A one-way ANOVA was applied to evaluate differences between the groups. Results: No implant fracture occurred during the artificial chewing simulation. Furthermore, there were no statistically significant differences (*p* > 0.05) between the groups in terms of fracture resistance (group 0: 783 ± 43 N; group H: 742 ± 43 N; group L: 757 ± 86 N; group HL: 740 ± 43 N) and bending moment (group 0: 433 ± 26 Ncm; group H: 413 ± 23 Ncm; group L: 422 ± 49 Ncm; group HL: 408 ± 27 Ncm). Conclusions: Within the limitations of the present investigation, it can be concluded that artificial loading and hydrothermal aging do not reduce the fracture resistance of the investigated implant system.

## 1. Introduction

Oral implants are applied for single tooth replacements, in partially edentulous as well as in edentulous scenarios to retain restorations. This treatment modality can increase patients’ satisfaction significantly. Due to their biocompatibility, corrosion resistance, mechanical properties, and long-term clinical success [1,2,3,4], titanium implants are regarded as the gold standard in oral implant dentistry.

Driven by the demand for the enhancement of long-term stability, titanium oral implants have been continuously improved since their introduction in the late 1970s/early 1980s [5,6,7]. An adaption of implantation protocols and drilling techniques was also performed [8,9].

Despite the progress and reported survival rates of 95% or higher over 5–10 years for titanium oral implants [10], the demand for metal-free alternatives is rising. On the one hand, concerns regarding the potential release of titanium particles exist [11], which may lead to titanium hypersensitivity or titanium allergy [12,13]. On the other hand, aesthetics may be hampered due to the grey color of the titanium, which might be problematic for patients with a thin gingival phenotype in the anterior regions [14]. To overcome these drawbacks with the use of titanium as an implant material, ceramic implants could provide a potential metal-free and aesthetic alternative. Nowadays, ceramic implants are fabricated from zirconia (zirconium dioxide ZrO_2_), most commonly from yttria-stabilized tetragonal zirconia polycrystal (Y-TZP). The stability of this material is increased by the phenomenon of allotropy, which enables a phase transformation toughening mechanism [15]. In addition to the increase in stability, further advantages of zirconia might include the reduction of biofilm formation in comparison to titanium implants [16] and better biocompatibility [17].

Regardless of the preclinical obtained positive characteristics of zirconia oral implants, to date there is only limited clinical data concerning the use of zirconia implants. Nevertheless, Pieralli et al. (2017) found in their systematic review of clinical applications of zirconia oral implants that for single-tooth replacements and three-unit implant-retained bridges, zirconia implants can be considered a treatment option with an outcome comparable to titanium implants [18].

Zirconia implants are available as one-piece implants, where the abutment is integrated into the implant, and two-piece implants, which require a connection of the abutment and the implant. So far, zirconia oral implants used in clinical investigations have been mostly of a one-piece design [19]. However, one-piece implants do not enable axis corrections and thus reduce prosthetic flexibility [20]. Furthermore, in case of a fracture of the transgingival part of the implant, the removal of the entire implant is necessary [21]. A possibility to avoid these disadvantages is the use of two-piece implants. They consist of an enossal part, the actual implant body, and a separate abutment attached in different ways to the implant body [18,19]. Adhesive cementation of abutments to zirconia implants has been described in the literature [22,23,24]. The technique-sensitive workflow, however, did not show satisfying results [23]. The disadvantages of cementing abutments can be overcome using a retrievable screw retention [25,26,27,28,29]. These laboratory investigations showed that some of the two-piece systems are stable enough to be applied clinically [25,26,27] whereas others might be not stable enough [28,29]. It seems advisable that new ceramic implant systems are evaluated biomechanically in in vitro studies before being brought to the market.

Therefore, the aim of the present investigation was to evaluate the fracture resistance of a market-available two-piece zirconia implant system with a carbon-reinforced polyetheretherketone (PEEK) screw for the retention of the abutment after exposure to artificial aging in an artificial chewing simulator. For the investigated implant system, no scientific in vitro data has been available so far.

## 2. Materials and Methods

### 2.1. Experimental Set-Up

Thirty-two market-available two-piece zirconia implants were divided into four groups (*n* = 8) and underwent different artificial loading and hydrothermal aging protocols in a chewing simulator. The specimens of group 0 were neither artificially loaded nor hydrothermally aged but evaluated for fracture resistance; group H was subjected only to hydrothermal aging and no artificial loading; group L was exposed to only to artificial loading and no hydrothermal aging; and group HL was subjected to hydrothermal aging and artificial loading. One specimen of each group was randomly selected, cross-sectioned, and analyzed regarding its crystal composition by scanning electron microscopy (SEM). With the exception of these specimens, all other specimens that survived the dynamic loading and aging underwent a quasi-static loading test in a universal testing machine until fracture. The results were statistically evaluated.

### 2.2. Experimental Implant

**Figure 1:**
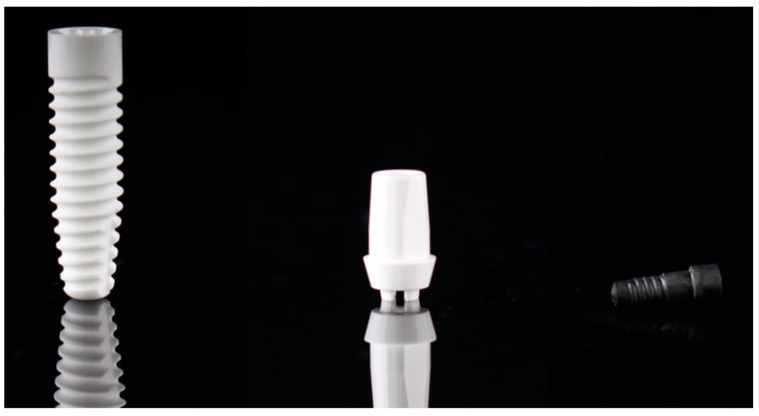
Experimental two-piece zirconia implant. **Left**: Zeramex XT implant; **middle**: Zeramex XT abutment; **right**: carbon-reinforced PEEK abutment screw.

The study was conducted with an alumina-toughened zirconia implant system (Zeramex XT, Dentalpoint AG, Spreitenbach, Switzerland, Figure 1). All implants had a length of 14 mm and a diameter of 4.2 mm. The implants were provided with a sandblasted (Al_2_O_3_-particle size: 105–140 μm) and acid-etched (H_3_PO_2_) implant surface (Zerafil; Dentalpoint AG, Sa = 0.7 μm, St = 9.6 μm, and Ssk −0.4644; wettability contact angle: 21° [30]). The upper implant collar part (0.6 mm), however, was not sandblasted and etched but showed a smooth surface. The implants were restored with zirconia abutments (Zeramex XT abutment straight; Dentalpoint AG) which had an emergence profile height of 2 mm and an anti-rotational protection. The implant and the abutment were connected with a carbon-reinforced polyetheretherketone (PEEK) screw (Vicarbo screw; Dentalpoint AG).

### 2.3. Preparation of the Specimens for the Artificial Loading/Hydrothermal Aging

**Figure 2:**
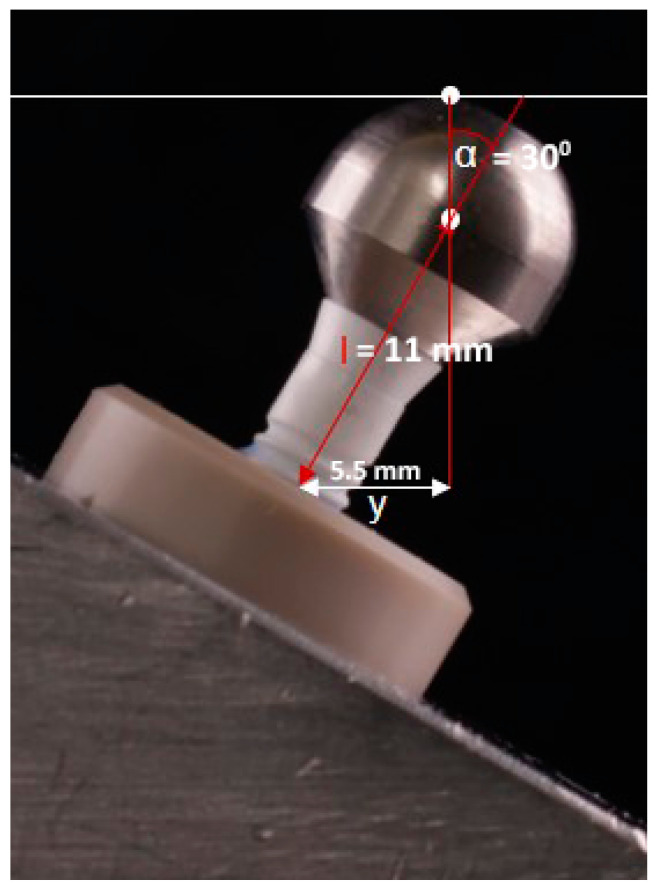
Specimen mounted according to the ISO 14801 standard. The investigated two-piece zirconia implant is restored with a loading sphere. I = the distance between the embedment plane and the loading center; α = loading angle; y = lever arm.

The implants were embedded in PEEK tubes (Figure 2) using a dual-curing acrylate-based resin (LuxaCore^®^ Dual, DMG, Hamburg, Germany) according to the ISO 14801 standard [31]. The PEEK tubes had an adjustable inner bottom for bringing the implant into the correct depth. The resin for embedding had a Young’s modulus of about 3.2 GPa and met the requirements of the ISO 14801 standard. All implants were embedded 3 mm above the artificial bone level (=upper rim of the PEEK tube) to simulate bone loss, as specified in ISO 14801. Subsequently, the abutments were tightened with a torque of 25 Ncm using a ratchet. A metal sphere with a diameter of 5.5 mm was attached to the abutment for force transmission to the implant–abutment assembly. The angle between the implant axis and the planned loading axis was 30 ± 2°. The distance between the implant exit from the tube/embedding resin and the loading center of the implant was 11 ± 0.5 mm. This installation resulted in a lever arm of 5.5 ± 0.5 mm.

### 2.4. Dynamic Loading and Hydrothermal Ageing

**Figure 3:**
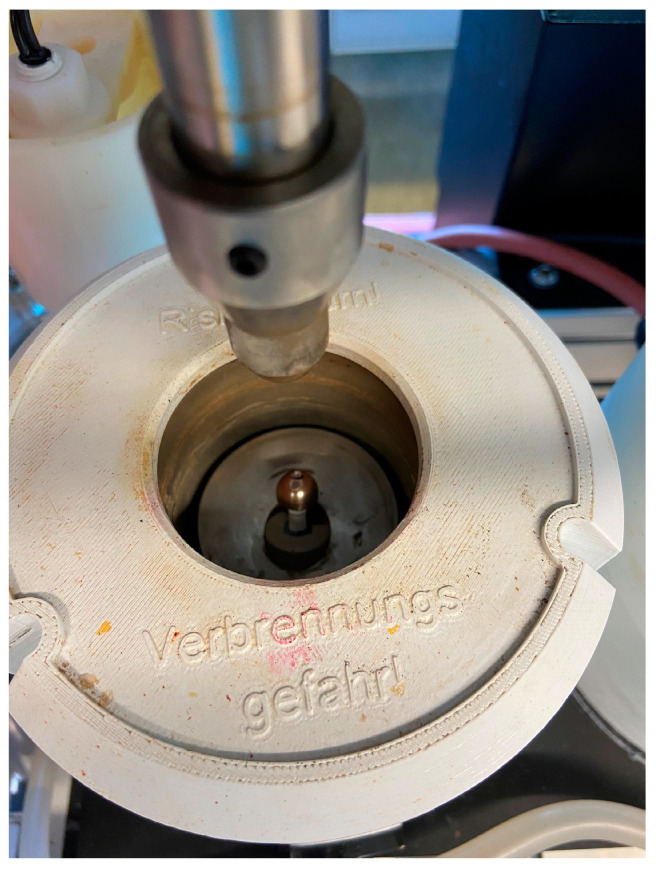
A specimen of group L (only exposed to artificial loading). Situation at the end of the loading interval.

All specimens were mounted into a computer-controlled dual-axis high-temperature chewing simulator (CS-4.8, SD-Mechatronik, Feldkirchen-Westerham, Germany; Figure 3) using special tube holders. The dynamic loading with a load of 98 N corresponding to a bending moment of about 54 Ncm was executed with a stainless-steel antagonist with a flat surface for 10 million cycles (frequency: 1.3 Hz). For hydrothermal aging, the specimen chambers were filled with distilled water, which was constantly heated to 85 °C through an integrated heating system. The evaporation of water was counteracted with an automatic refill system. One chewing cycle was composed of a vertical load (60 mm/s) and a subsequent horizontal movement of 0.5 mm (55 mm/s) to mimic physiological mastication. As mentioned above, the implants of group H were only exposed to hot water; no loading was executed. The implants of group L, hence, were only loaded and not hydrothermally aged. The samples from group HL were subjected to simultaneous hydrothermal treatment and dynamic loading. During the testing intervals, the specimens were evaluated twice a day for fractures and abutment loosening.

### 2.5. Scanning Electron Microscopy (SEM) of Cross-Sectioned Specimens

**Figure 4:**
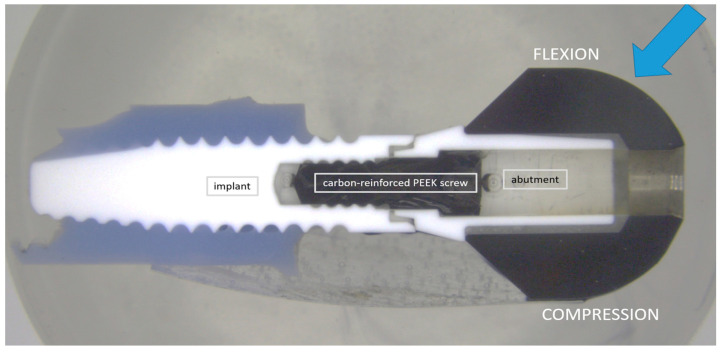
Evaluated regions in the SEM analysis of the implant and abutment. Transformation propagation was analyzed at the outer surfaces of the abutment and implant and in the region where the abutment connected to the implant shoulder. The blue arrow indicates the loading direction of the samples of groups L and HL resulting in a flexion and compression side, analyzed separately for these two groups.

For the SEM evaluation, one of the eight specimens from each group was fixed in epoxy resin (EpoFix, Struers, Ballerup, Denmark) and then bisected on a precision sectioning saw with a 0.5 mm bronze-bonded diamond-sawing blade (Figure 4). The implant was inserted in a fixture and the contact between the diamond cutting blade on the left and right side of the implant allowed the center of the implant to be identified. To prepare the cross-section of the implant close to the center, the cut was carried out with water cooling at a position slightly offset from the center to compensate for the thickness of the blade (0.5 mm) and the material removal during final polishing. The blade speed was set to 150 rpm and a constant load of approximately 50 g. After grinding the bisected samples, they were polished using diamonds and a silica suspension (Struers Tegramin-30, Struers). After coating the specimens with carbon, they were examined using a field emission scanning electron microscope (Jeol JSM-7800F, Akishima, Japan). The crystal transformation zone of the zirconia material was evaluated using the SEM images. The average size of the grains was measured with an image analysis software (Adobe Photoshop CS6, Adobe Inc., San José, CA, USA). Approximately 150 randomly selected grains from each phase were measured. The average grain sizes of ZrO_2_ and Al_2_O_3_ were determined by applying the linear intercept method using 1.56 as the correction factor [32,33].

### 2.6. Static Loading Test

**Figure 5:**
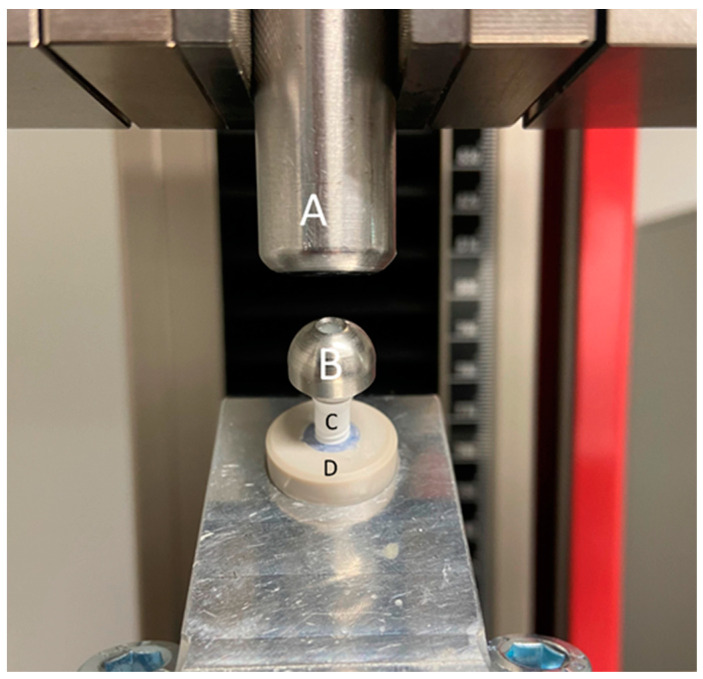
A specimen inserted in the universal testing machine (ZwickRoell Z010) at an angle of 30° to the loading axis. A: the metal antagonist; B: the loading sphere; C: the two-piece zirconia implant; D: the PEEK tube.

For testing the fracture resistance, seven of the eight specimens from each group were inserted in a universal testing machine (ZwickRoell Z010, ZwickRoell AG, Ulm, Germany) at an angle of 30° to the loading axis and were then loaded to fracture (Figure 5). The crosshead speed of the testing machine was set to 10 mm/min [27,34,35], and the endpoint of loading was defined at the sudden drop in load capacity. The fracture patterns were visually evaluated and defined as a partial or complete fracture of the implant/abutment (Figure 5).

### 2.7. Statistical Analyses

For statistical analyses, a single factor variance analysis (one-way ANOVA) was applied to determine the differences in fracture load and bending moment between the study groups. For subsequent pairwise comparisons, the Student–Newman–Keuls method was conducted to correct for multiple testing. Paired *t*-tests were applied for the determination within groups. The level of statistical significance was set to *p* < 0.05. The calculations were performed with a statistical analysis software (STATA 17.0, StataCorp LP, College Station, TX, USA).

## 3. Results

### 3.1. Dynamic Loading Test

The test specimens were dynamically loaded with F = 98 N (10 kg), which resulted in a bending moment of 54 Ncm. All specimens survived 10 million cycles of dynamic loading and hydrothermal aging at 85 °C without fractures, other defects, or loosening of the abutment screw (100% survival).

### 3.2. Scanning Electron Microscopy (SEM)

**Figure 6:**
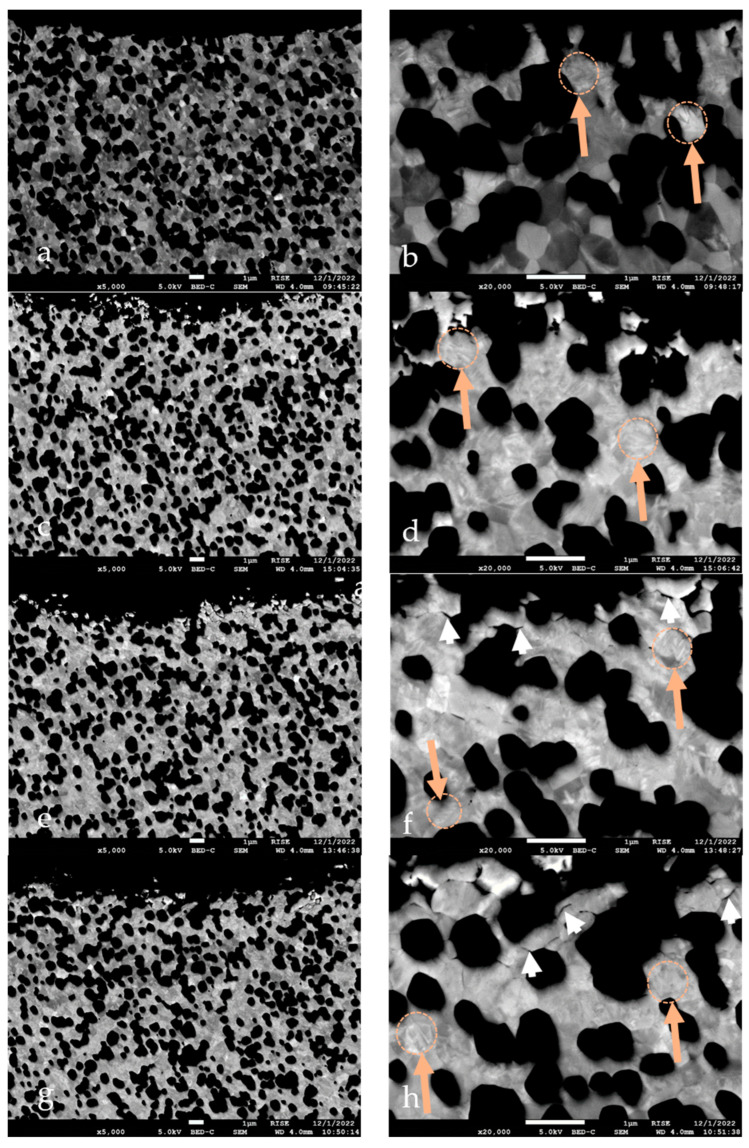
All SEM images present the implant surface area from the flexion site. The left picture column depicts the areas at a magnification of 5000 and the right at a magnification of 20,000. (**a**,**b**) an implant from group 0. The alumina grains are black, and the ZrO_2_ grains are in brighter colors. No clear transformation boundary can be depicted in any situation. (**c**,**d**) an implant from group L. (**e**,**f**) an implant from group H. (**g**,**h**) an implant from group HL. Intergranular microcracks are shown with white arrowheads and transformation of grains with twinning in orange arrows and dotted circles.

Since no differences in transformation patterns occurred between the different areas of evaluation, only the implant surfaces of the flexion site were presented in SEM figures (Figure 6a–h). The SEM images revealed the two phases of the material. The brighter grains were identified as yttria-stabilized ZrO_2_ and the dark grains were Al_2_O_3_. The average grain size of the Al_2_O_3_ grains was 0.5 ± 0.1 µm. The grain sizes between the different treatments did not differ. In addition, no clear t-m transformation zone could be observed in the SEM figures (Figure 6a–h). However, after loading, hydrothermal aging, and combined treatment, transformation effects were more often visible in the zirconia grains (Figure 6c–h). Furthermore, the loaded, aged, and loaded-aged specimens showed intergranular microcracks at and slightly below (~2 µm) the implant surface.

### 3.3. Static Loading Test

The mean load to fracture was 783 N (SD: 43 N) for the control group 0, 742 N (SD: 43 N) for group H, 757 N (SD: 86 N) for group L, and 740 N (SD: 51 N) for group HL (ANOVA: *p* > 0.05). The mean bending moments that led to fracture amounted to 433 Ncm (SD: 26 Ncm) for group 0, 413 Ncm (SD: 23 Ncm) for group H, 422 Ncm (SD: 49 Ncm) for group L, and 408 Ncm (SD: 27 Ncm) for group HL (ANOVA: *p* > 0.05) (Table 1).

## 4. Discussion

The aim of the present in vitro study was to investigate the fracture resistance of a two-piece zirconia implant system connected with a carbon-reinforced PEEK abutment screw after exposure to hydrothermal aging and loading. The key findings of the present investigation were that hydrothermal aging and loading did not influence the fracture resistance of this implant system. Furthermore, the results of our investigation indicated that the stability of the tested system is high enough to withstand long-term normal intraoral occlusal forces [36]. The fracture resistance of a zirconia implant should endure the maximum voluntary bite forces of patients, and the highest bending moment while biting was measured in vivo as 95 Ncm [37]. When a safety margin of 100% is applied, a minimum fracture resistance of 200 Ncm should be sufficient for clinical safety [36]. This minimum fracture resistance was found for all our tested groups.

All specimens of groups L and HL survived the dynamic loading test of 10 million cycles with a load of 98 N (=54 Ncm) without visible defects at the implants/abutments and without the loosening of the implant–abutment connections. The application of ten million chewing cycles was frequently described in the literature when investigating the fracture resistance of zirconia oral implants [27,38]. Due to heterogeneous results from investigations evaluating chewing contacts per year [39,40], the 10 million chewing cycles used in the present study corresponded to a clinical timeframe ranging from 12.5 [40] up to 40 years [41,42,43,44].

To be comparable to other in vitro investigations evaluating zirconia implants, the implants in the present investigation—as in others—were embedded following the ISO 14801 standard. To imitate the clinical situation, an acrylate-based resin with an elastic modulus of approximately 3 GPa, which was similar to the elastic modulus of human bones, was utilized [45]. Furthermore, a peri-implant bone loss of 3 mm was simulated, although this value is much higher than the peri-implant bone loss observed in recent clinical investigations [46,47].

Artificial aging can induce spontaneous transformations from the metastable tetragonal to the monoclinic phase. This process is described as low-temperature degradation [48,49]. Even though the exact mechanism of low-temperature degradation (LTD) is not fully understood, it seems that water molecules are incorporated into the polycrystal structure. The water derivatives cause the tetragonal to monoclinic transformation leading to a volume increase of 4% [50]. Each expanding grain causes stress on the surrounding material and leads to microcracking and further water penetration [51]. Eventually, this may lead to a decrease in the mechanical long-term behavior of zirconia in the oral cavity. Since zirconia oral implants manufactured from Y-TZP are prone to LTD, zirconia–alumina composites such as alumina-toughened zirconia are not [52].

Alumina-toughened zirconia thus seems to be a favorable material for zirconia implant fabrication [53] and was used in our in vitro set-up where hydrothermal aging at 85 °C was performed, which is in accordance with other investigations [27,35]. The intention was to evaluate the impact of hydrothermal aging on phase transformation as was conducted previously on pure 3Y-TZP zirconia oral implants [35]. The crystal structure in pure 3Y-TZP material is usually well-ordered and the slight variation in the contrast that allowed the individual grains to be identified in microscopic images was assumed to be due to the crystal orientations in the individual grains. When the aging caused the tetragonal grains to transform into a monoclinic structure, the phase transformation also induced an internal disorder of the crystals. This contributed to an increased contrast between the tetragonal and monoclinic grains. The layer of monoclinic zirconia grains at the surface exposed was then clearly distinguishable from the tetragonal phase in the bulk of the material, which allowed the thickness of the transformed layer to be determined. However, in alumina–zirconia composites, the tetragonal zirconia was found to have a similar degree of crystal disorder in both the tetragonal and the monoclinic phases. Therefore, the change in contrast earlier obtained from the phase transformation in pure 3Y-TZP did not occur in the alumina–zirconia composite. The thickness of the layer where the tetragonal grains were transformed to monoclinic grains was then not clearly detectable from the contrast in the images. The crystallographic phase change further resulted in a volume increase that caused microcracks around the transformed zirconia grains, which allowed the thickness of the layer with transformed zirconia grains to be identified. In the HL group, microcracks were found to be present to a depth from the surface of around 2 microns, in the L group to a depth of around 1 micron, while almost no indication of transformation was detected in the H and 0 groups.

In their in vitro investigation of mostly experimental two-piece zirconia implants, Preis et al. (2016) found that the type of connection between implant and abutment is crucial for the resistance to failure [54]. Many of the two-piece zirconia implants where the abutments have been connected to the implant via a screw (either titanium or carbon-fiber-reinforced polymer) already failed during thermo-cycling and mechanical loading leading to low forces to fracture. The present market-available implant system with the carbon-fiber-reinforced PEEK screw showed no failure of the implants, abutments, or screws after artificial loading, which is in contrast to the results of Preis et al. (2016) [54].

Carbon-fiber-reinforced PEEK screws enable a metal-free approach for patients and are therefore not prone to corrosion and the subsequent risk of fatigue fracture. Furthermore, there is a smaller risk for the abrasion of the inner implant threads due to their lower hardness [55]. Lastly, a screw removal in case of screw fracture is described as simpler compared to titanium screws [56]. Nevertheless, in vivo investigations are necessary to evaluate the clinical performance of zirconia implants in combination with the carbon-fiber-reinforced PEEK screws.

The results of the quasi-static loading test after chewing simulation in the present investigation showed bending moment values that lead to fatal failure for the different scenarios between 407 and 432 Ncm. The highest values were found in the control group without loading, whereas the group with hydrothermal aging and loading presented the lowest values. Nevertheless, these values were manifold higher than the range of clinically measured bending moments of 5–95 Ncm with strain gauges at implant-supported fixed partial dentures [37]. Based on these results, the clinical safety of this system could be expected.

There is only limited preclinical and clinical data available on two-piece zirconia implants [18,19,36]. Due to this lack of scientific data for two-piece zirconia implants, Roehling et al. (2018) stated that “1-piece zirconia implants can be considered as a reliable treatment option for follow-up periods up to 2 years. Regarding the clinical application of 2-piece zirconia implants, very little evidence-based data are available” [19].

Nevertheless, the advantages of two-piece zirconia implants would be the same as for two-piece titanium implants. Submerged healing in combination with a guided bone regeneration procedure might be regarded as advantageous over the non-submerged one-piece implants. The possibility of retrievability of screw-retained superstructures for reasons of repair and implant cleaning also might be seen as an advantage. Furthermore, the possibility to use angled abutments in case of a suboptimal implant position as well as the prevention of cement entrapment while using screw retention may be listed as positive aspects.

Nevertheless, clinical trials on the overall performance of two-piece zirconia implants are needed to identify potential technical or biological complications.

## 5. Conclusions

Within the limitations of the present investigation, it can be concluded that artificial loading and hydrothermal aging do not reduce the fracture resistance of the investigated implant system.

## Figures and Tables

**Table 1:** Mean values and standard deviation of the load (N) and bending moment (Ncm) of all groups.

Group	Fracture Resistance (N)	Bending Moment (Ncm)
0	783 ± 43	433 ± 26
H	742 ± 43	413 ± 23
L	757 ± 86	422 ± 49
HL	740 ± 51	408 ± 27

Group 0 = not loaded and not aged; group H = only hydrothermally aged; group L = only loaded; group HL = aged and loaded. No significant differences occurred between the groups regarding fracture strength and bending moment.

## Data Availability

The data presented in this study are available on request from the corresponding author.
